# Optical Fiber Sensors for Ultrasonic Structural Health Monitoring: A Review

**DOI:** 10.3390/s21217345

**Published:** 2021-11-04

**Authors:** Rohan Soman, Junghyun Wee, Kara Peters

**Affiliations:** 1 Institute of Fluid Flow Machinery, Polish Academy of Science, 80-231 Gdansk, Poland; 2 Department of Mechanical and Aerospace Engineering, North Carolina State University, Raleigh, NC 27695, USA; junghyunwee10@gmail.com (J.W.); kjpeters@ncsu.edu (K.P.)

**Keywords:** structural health monitoring, fiber optic sensors, ultrasonic, guided waves, acoustic emission

## Abstract

Guided waves (GW) and acoustic emission (AE) -based structural health monitoring (SHM) have widespread applications in structures, as the monitoring of an entire structure is possible with a limited number of sensors. Optical fiber-based sensors offer several advantages, such as their low weight, small size, ability to be embedded, and immunity to electro-magnetic interference. Therefore, they have long been regarded as an ideal sensing solution for SHM. In this review, the different optical fiber technologies used for ultrasonic sensing are discussed in detail. Special attention has been given to the new developments in the use of FBG sensors for ultrasonic measurements, as they are the most promising and widely used of the sensors. The paper highlights the physics of the wave coupling to the optical fiber and explains the different phenomena such as directional sensitivity and directional coupling of the wave. Applications of the different sensors in real SHM applications have also been discussed. Finally, the review identifies the encouraging trends and future areas where the field is expected to develop.

## 1. Introduction

Structural health monitoring (SHM) systems are necessary in structures in order to detect any deterioration in the structure and avoid catastrophic failures. In addition, SHM systems can minimize maintenance costs and the down-times of critical structures, thus allowing a very high return on investment [1]. As a result, SHM systems are receiving great interest from the scientific community [2,3]. SHM can be loosely defined as the assessment of the condition of the structure through the monitoring if its in-service performance. Typically sensors used to detect vibration, strain, corrosion, etc., are placed on the structure and through monitoring damage sensitive features the condition of the structure can be determined. A wide range of SHM techniques have been developed over the last few decades, which make use of several different technologies and damage sensitive features [4,5,6,7].

SHM techniques based on vibration and strain have been more popular in the civil engineering sector [8,9], while the aerospace sector has been active more in the use of acoustic emission (AE) -based techniques and elastic wave propagation [10,11]. The focus can be explained on the basis that the vibration-based techniques tend to be global damage detection techniques, which are suitable for detecting high levels of damage that are acceptable in the civil engineering, as civil engineering structures are more damage-resilient. In the aerospace sector, composites are commonly used and are not as damage tolerant as metals. The use of composites due to their greater strength to weight ratio as well as resistance to harsh conditions is increasing. As a result, more work in the area of SHM with a focus on the reliable detection of small levels of damage, as well as the ability to not only detect but also localize and diagnose the damage, is necessary. For small levels of damage, ultrasonic-based techniques such as guided waves (GW) and AE have been very popular [12,13] and as a result are a focus of this paper.

A typical GW-based SHM system requires the use of sensors for data collection, filters for data cleansing, and central data processing units for feature extraction and post processing. The current paper focuses on the sensors used for the data collection. Optical fiber-based sensors offer several advantages as compared to other sensors and have been extensively used for SHM. These benefits can be summarized as [14]:

(i) Low power loss;
(ii) Resistance to electromagnetic interference;
(iii) Low security risk;
(iv) Small size;
(v) Light weight;
(vi) Large bandwidth accommodation;
(vii) Resistance to harsh conditions.

The optical fiber is sensitive to the outside stimulus and the environment, which affects the light propagating through the fiber in terms of frequency, phase, amplitude, polarization, and intensity. These changes in the properties can be measured and calibrated to form many kinds of sensors. Fiber optic (FO) sensors have been used for the measurement of rotation, velocity, displacement, torque, acceleration, humidity, chemical presence, temperature, pressure, etc [15]. The optical fibers also provide capabilities to the sensors, which are not always possible by conventional measurement techniques. For instance the strain measurement capabilities of FO sensors allow distributed measurement over a wider gauge length (up to a few meters) while traditional strain gauges are essentially point sensors. Furthermore, some FO-based sensors allow simultaneous measurement of temperature and strain, which allows for easy compensation of the effects of ambient condition changes. Lastly, optical fiber-based sensors also have faster sampling rates and hence better time resolution, which is also a desirable feature. The ability of the FO sensors to detect mechanical and dynamic properties has allowed their use for SHM [10].

The paper provides an overview of the state of the art of GW-based SHM and AE-based SHM techniques, which have been developed with the use of the novel FO-based sensors. It should be noted that the aim of the paper is not a comparative study of the different types of sensors, nor is it the comparison of different processing techniques used. The techniques that have been reported in the literature have been tailored for specific applications, and for the optimal use of the techniques, fine-tuning is necessary. This paper tries to present the state of the art in the use of FO sensors for ultrasonic-based SHM techniques. Two review papers that focused on FBG sensors for ultrasonic measurements [16,17] have recently been published. Although these review papers cover a lot of papers in the field, they do not provide an overview of sensors other than FBG sensors. Additionally, for the FBG sensor-based systems, they do not address more recent advances and the physics of the measurement of the GW using FBG sensors in detail. They do not cover the physical phenomena such as the directional sensitivity of the FBG sensors, the different mechanisms of the coupling of the wave to the fiber as well as the different transduction mechanisms for measuring waves with different wavelengths. Hence, there is a need for an in-depth review that brings these phenomena to the attention of researchers working in this fast-growing field.

The paper is organized as follows. The next section discusses the classification of the FO sensors based on the principle of operation and explains the principles of FO sensors which have been used for GW-based measurement. The following section focuses on fiber Bragg grating sensors for GW sensing. Section 4 covers the SHM applications of FO sensors. In the final section, some areas of future work and the potential problems have been identified by the authors.

## 2. Classification of FO Sensors

**Figure 1:**
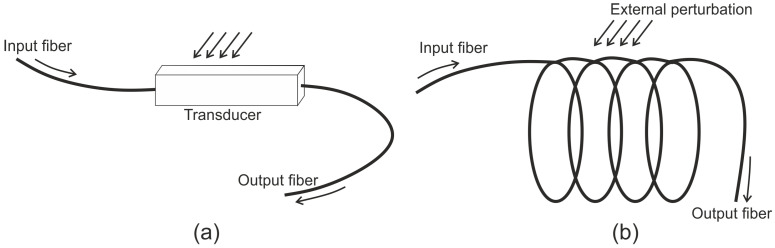
Fiber-optic sensor types: (**a**) extrinsic and (**b**) intrinsic (based on reference [18]).

The broadest classification of the FO sensors is into intrinsic sensors and extrinsic sensors. For extrinsic sensors (Figure 1a), the optical fibers serve as a delivery mechanism in order to carry the light to the sensing region, which is outside of the fiber. The light is then affected by the physical environment and the optical fiber carries the modulated response to a detector for processing. For intrinsic sensors, as shown in Figure 1b, the optical fiber works as a communication medium as well as the transducer.

The earliest FO sensor, an extrinsic sensor, was developed and patented in 1967 as a proximity probe. The development of the intrinsic sensor can be traced back to Davies et al. [19], who observed the effect of mechanical strain on the light passing through the fiber. The possibility of having a continuous medium and the ease of the intrinsic use attracted a lot of further interests in the area and resulted in several different types of sensors, making use of a wide range of operating principles.

The modulation of the light based on physical conditions gives us another way of classifying the sensors. The FO sensors can be classified into

(i) Scattering-based sensors;
(ii) Intensity-based sensors;
(iii) Polarization-based sensors;
(iv) Phase-based sensors;
(v) Wavelength-based sensors.

### 2.1. Scattering-Based Sensors

**Figure 2:**
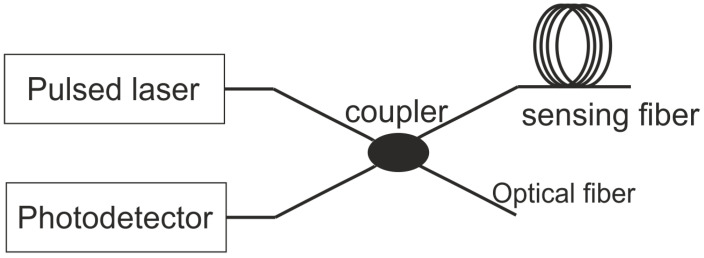
Schematic of ϕ OTDR (based on reference [21]) .

Scattering-based sensors include sensors based on Rayleigh, Raman, and Brillouin scattering principles. When a short-pulsed light is launched into the optical fiber, most of the light travels through the fiber, while small portions of the incident light are scattered back along the optical fiber. The back-scattered light can be categorized into the three wave bands mentioned above. Rayleigh scattering has the same wavelength as the launched pulse light, whereas both Brillouin and Raman scattering are shifted in wavelength. All three phenomena can be used for the strain and temperature monitoring along the entire length of the fiber [20]. The distributed acoustic sensing (DAS) systems were developed mainly using the phase sensitive optical time domain reflectometry (ϕ OTDR) and optical frequency domain reflectometry (OFDR). (ϕ OTDR) is more commonly used for acoustic sensing as it is capable of a higher sampling rate for the DAS systems. The ϕ OTDR system makes use of a single frequency laser pulse and a photodetector, as shown in Figure 2. The external perturbation changes the propagation phase of the probe pulse. The change in the phase Δϕ is due to the change in length as well as the change in the refractive index and is given by Equation (1)

(1) $$\Delta \phi =\left(1+\gamma \right)\eta_{0}kl\epsilon$$

where, γ is the elasto-optical coefficient and $\eta_{0}$ is the refractive index.

The frequency with which the pulse can be repeated depends on the fiber length. The time needs to be longer than the time required for the propagation of the light to the end of the fiber and back. The repetition rate in turn determines the sampling rate of the system. Out of the three scattering principles, the Rayleigh scattering is the most commonly used because of the higher signal strength [22]. Although the distributed nature and the large range are both highly desirable, the sampling rate achieved by these sensors is very limited. It depends on the length of the sensing fiber. A maximum of 100 kHz has been realized, but for higher fiber lengths the sampling rate is restricted to a few kHz (for 10 km around 10 kHz is possible, and for 50 km around 2 kHz is possible). For Raman and Brillouin scattering, several averages over several pulses are necessary to obtain a reliable measurement due to the lower signal strength. As a result, the sampling rate is limited to several Hz. The sampling frequency achieved using the Raman and Brillouin scattering effects as well as the OFDR technique is far too low for their use in ultrasonic SHM and hence these techniques are considered beyond the scope of this paper [23,24,25]. The interested readers are referred to [26] for the details about these methods.

### 2.2. Polarization-Based Sensors

When a light-wave propagates along the optical fiber, its polarization state can change due to the difference in the phase velocity of the two polarization components in a birefringent fiber. The polarization properties of light propagating through an optical fiber are sensitive to external conditions such as stress, strain, pressure, and temperature. This sensitivity is harnessed in a fiber polarimetric sensor to retrieve the sensing parameter based on the change in the polarization [27]. Cross sensitivity between different external parameters is a major challenge to these types of sensors. The capability of polarimetric fiber sensors to measure strain, temperature, and pressure is demonstrated in a variety of applications including for SHM [28]. This sensor is popularly used as a hydrophone for acoustic measurements. However, the sensitivity of the polarization state to low amplitude ultrasonic structural waves is low [29]. This is primarily due to the fact that a large amount of birefringence in the optical fiber must be induced to produce measurable changes in the polarization state. For a surface mounted sensor, the loading is only applied along one side of the optical fiber and the transverse pressure is not near that induced by a loading state such as hydrostatic loading. Additionally, methods based on birefringence do not make use of any systematic amplification (such as the high slope of the FBG sensor in edge filtering).

### 2.3. Intensity-Based Sensors

Microbend sensors are the most popular example of intensity-based sensors. Any perturbation in the structure introduces small microbends in the optical fiber [30]. These microbends result in a change in the intensity of the transmitted light, which may be monitored for detecting the perturbations [31]. This phenomenon can be used for detecting a propagating wave or acoustic event. However, the amplitude of guided waves are extremely small and are again only applied to one side of the optical fiber for surface mounted sensors. The resulting bending and therefore bending loss in the optical fiber is extremely low. Therefore, microbend sensors are unsuitable for GW monitoring.

**Figure 3:**
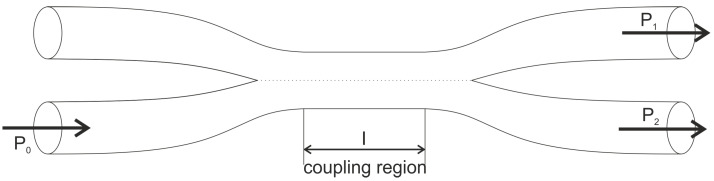
Schematic of fused tapered coupler (based on reference [34]) .

These challenges with microbend sensors have been overcome through the use of fused tapered couplers. In this sensor (Figure 3), two tapered optical fibers are fused together using a high temperature flame [32]. This process brings the core region of the two optical fibers in contact, which in turn allows the light transfer across the tapered region [33]. For the input energy $P_{0}$, the output energy in the two output ports is given by Equation (2) [34].

(2a) $$\begin{array}{cc} \;\;\;\;\;\;P_{1} & =P_{0}\sin^{2}\left(\int_{-l/2}^{l/2}C\left(z\right)dz\right), \end{array}$$

(2b) $$\begin{array}{cc} \;\;\;\;\;\;\;\;\;\;P_{1} & =P_{0}\sin^{2}\left(Cl\right),\;\;\mathrm{if}\;C\;\mathrm{is}\;\mathrm{constant}, \end{array}$$

(2c) $$\begin{array}{cc} P_{2} & =P_{0}-P_{1} \end{array}$$

where *C* is the coupling coefficient, and *l* is the coupling length. When the coupled region is subjected to an excitation, the coupling ratio changes, leading to a change in the output power at each port. By measuring the output power, the incident wave can be measured. The tapered region works as a amplifier for the output intensity to the applied strain. Fused couplers are easy to manufacture [35,36,37] and can be easily embedded in the structure [38,39]. The tapered region is unfortunately very fragile and not often suitable for field applications.

### 2.4. Phase-Based Sensors

Phase- and wavelength-based sensors have a higher sensitivity to ultrasonic waves. Many different configurations have been used for ultrasonic sensing, several of which are discussed in the next two sections. Phase-based sensors are based on the principle of interferometry. They are among the most sensitive of the the fiber optic sensors. There are several different configurations of phase-based sensors. Some of the most important ones are:

(i) Fabry-Perot Interferometers (FPI);
(ii) Mach Zehnder Interferometers (MZI);
(iii) Michelson Interferometers (MI);
(iv) Sagnac Interferometers (SI);
(v) Twyman–Green Interferometers (TGI);
(vi) Rayleigh Interferometers (RI).

There are several other modifications of these configurations which have found applications in diverse fields. However, for GW sensing only the top four namely, FPI, MZI, MI, and SI, have been used and will be discussed in detail here.

**Figure 4:**
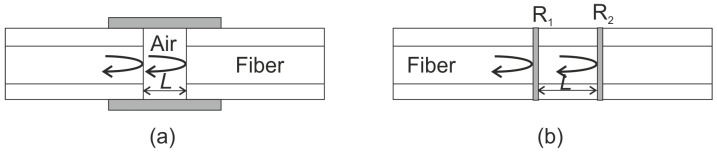
Principle of FPI (**a**) extrinsic (**b**) intrinsic (based on reference [47]).

An FPI (Figure 4) is generally composed of two parallel reflectors separated by a certain distance (cavity length). Both extrinsic [40,41] and intrinsic [42] configurations may be used for AE detection. The first use of FPI sensors as AE sensors was shown in 1978 by Bucaro and Carome [43]. The reflectors may be in the form of mirrors [44], interfaces between two dielectrics [45], or even Bragg gratings [42,46]. Interference occurs due to the multiple super-positions of both reflected and transmitted beams at the two parallel reflectors. The reflected and transmitted spectra of such an interferometer are functions of cavity length, index of refraction of the medium, and reflectivity of the mirrors.

**Figure 5:**
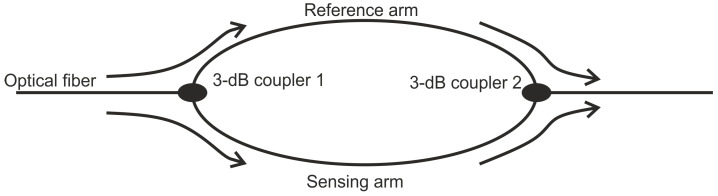
Principle of MZI (based on reference [49]).

The earliest MZI’s consisted of two arms—a reference and signal arm. The reference arm is unaffected by the outside stimulus while the signal arm is sensitive to the strain or temperature [48]. Due to the external parameters, a phase change is introduced between the signal and the reference arms. Splitting and recombination of the lightwave are performed at optical fiber couplers, as shown in Figure 5.

In the later applications, in-line MZI configurations have been used [50,51]. In the in-line configurations, a portion of the core mode of a single mode fiber is uncoupled from the fiber core mode into another mode and then re-coupled to the core mode by two interfaces or other elements. This approach allows the sensor to be compact. Additionally, having the two arms in-line allows ease of fabrication.

**Figure 6:**
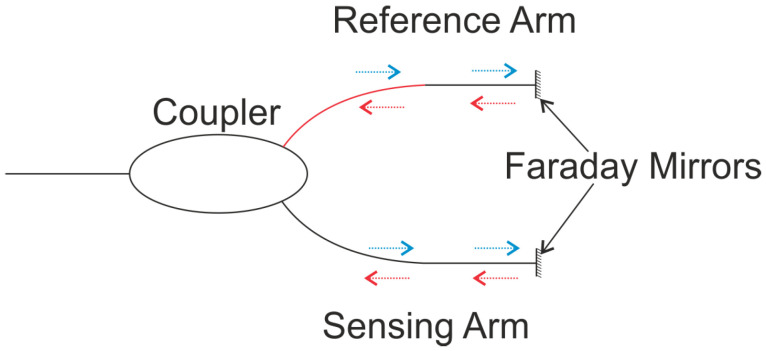
Principle of MI (based on reference [52]).

MI operation is based on the interference the lightwaves in the two arms, each reflected from mirrors placed at the end of the arm, as shown in Figure 6. The use of this configuration increases the compactness of the interferometer and allows multiplexing. In more advanced configurations, the generic light source may be replaced by a low coherence source, which allows the elimination of the phase ambiguity and the reflection-based problems.

**Figure 7:**
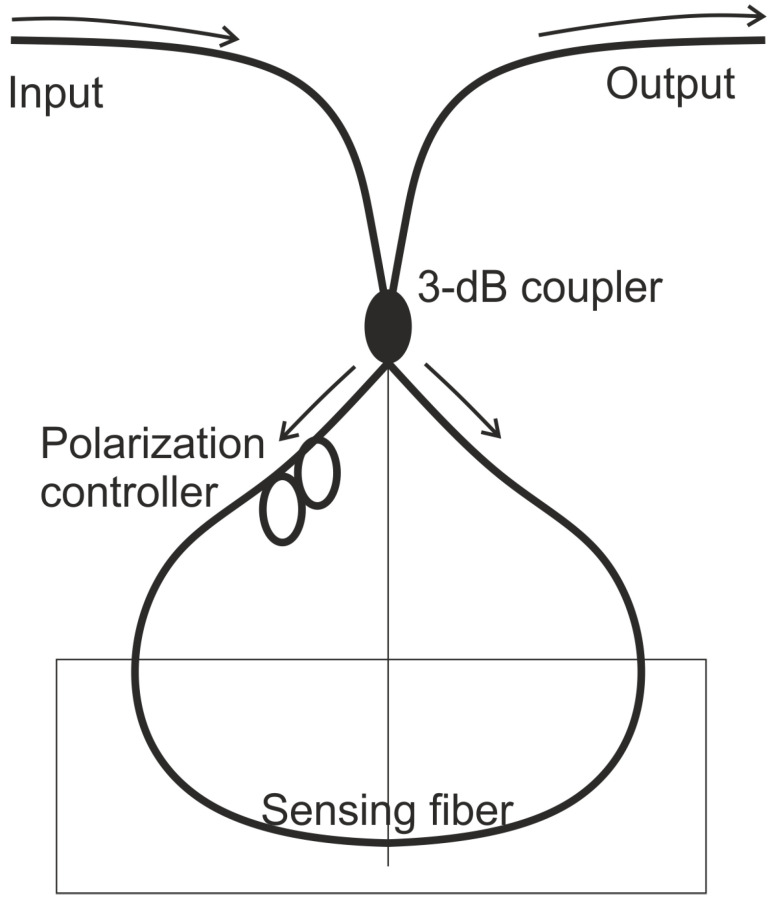
Principle of SI (based on reference [53]).

Sagnac Interferometers (SI) are also phase-based sensors, but the optical path length difference is determined by the polarization-dependent propagating speed of the mode guided along the loop. As with other interferometers, the light is split using a coupler. A loop is included in the sensing arm to achieve a configuration where the two counter propagating beams are combined in the same coupler, as shown in Figure 7. The polarizations are adjusted by a polarization controller attached at the beginning of the sensing fiber. The signal at the output port of the fiber coupler is governed by the interference between the beams polarized along the slow axis and the fast axis. The benefit of the SI is that the sensitivity for different applications can be customized by use of different fibers. Another benefit is the potential for simultaneous sensing capability with the help of other fiber optic devices.

In general, the sensitivity of interferometric sensors is very high and hence may be used for ultrasonic measurements. However, these sensing techniques are susceptible to static temperature or strain change along all the optical fibers [54]. This drift leads to the requirement of an expensive feedback controller and difficulty in measuring the time of arrival [55]. In addition, these sensors lack robustness in harsh environments, apart from low coherence configurations, and hence their use for ultrasonic SHM is limited to laboratory settings and more in the bio-medical industry where the conditions can be more controlled. Their applications in civil and mechanical engineering sectors are limited.

### 2.5. Wavelength-Based Sensors

**Figure 8:**
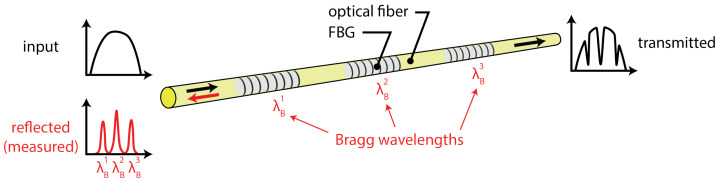
Operating Principle of FBG.

The most popular wavelength modulated sensors are grating-based sensors, such as the fiber Bragg grating (FBG). A fiber optic grating is formed by inducing a periodic refractive index perturbation along the length of an optical fiber core [56]. The grating works as a selective filter and reflects a selected wavelength while the rest of the incident light passes through. The operating principle for the FBG is shown in Figure 8. Whenever the environmental measures affect the grating region, it shifts the reflected peak wavelength. FBGs are point-like sensors with a small gauge length. A large number of FBGs can be multiplexed, because typical FBG spectral bandwidths are less than 1 nm.

The peak reflected or Bragg wavelength ($\lambda_{B}$) for the sensor is given by Equation (3) [57],

(3) $$\lambda_{B}=2n_{eff}\Lambda_{0}$$

where $n_{eff}$ is the effective refractive index of the fundamental core mode and $\Lambda_{0}$ is the grating period. When an FBG undergoes tension or compression due to the mechanical or thermal loads, its Bragg wavelength shifts. The Bragg wavelength shift ($\Delta \lambda_{B}$) is given by Equation (4) [58],

(4) $$\Delta \lambda_{B}=\lambda_{B}\left(\alpha +\zeta \right)\Delta T$$

where α is the thermal expansion coefficient, ζ is the thermo-optic coefficient, and ΔT is the change in temperature.

Due to their ease of use, robustness, and multiplexing ability, FBG sensors have been extensively used for the ultrasonic measurements. Traditionally, FBG sensors have been multiplexed using wavelength division, where a broadband light source or tunable light source is used. The Bragg wavelength is reflected from the FBG sensor, and the change in the reflected wavelength is determined by use of a spectrum analyzer or photodetector. Although the measurements in this configuration are highly reliable for static and low frequency vibrations, the sensitivity and the sampling frequency are not sufficient for ultrasound measurements.

In order to improve the sensitivity, the high reflectivity slope of the FBG sensor is often utilized to change the intensity or the total energy or the system output. An excellent description of different edge slope configurations can be found here [17]. In the power-based approach, a broadband laser followed by an optical filter is used to focus the light wavelength to have a overlap with the reflected spectrum of the FBG. The reflected power is detected by the photodetector, which integrates the voltage over the sensitive wavelength range. If there is a change in Bragg wavelength ($\lambda_{B}$), the the output voltage changes as Equation (5) [59],

(5) $$V\left(\Delta \lambda_{B}\right)=\int R_{D}\left(\lambda \right)S\left(\lambda \right)F_{filter}\left(\lambda \right)F_{sensor}\left(\lambda -\Delta \lambda_{B}\right)d\lambda$$

where, *V* is the voltage change as a function of $\Delta \lambda_{B}$, $R_{D}\left(\lambda \right)$ is the response factor of the photodetector, S(λ) is the power spectral density of the broadband power source, $F_{filter}$ and $F_{sensor}$ are the spectra of the filter and sensor, respectively, and λ is the wavelength in the range of interest. Different variations of this modulation approach have been used in the literature. The optical filter may be achieved through the use of matched FBG filters [60], microelectromechancial tunable filter [61], an FBG with a narrow full width half maximum (FWHM) [62], Mach Zehnder interferometer [63], or arrayed wave guide grating [64]. The sensitivity of the demodulation technique is proportional to the slope of the FBG sensors. The biggest advantage of this demodulation technique is its ability to be multiplexed, but the sensitivity is barely sufficient for ultrasonic measurements.

**Figure 9:**
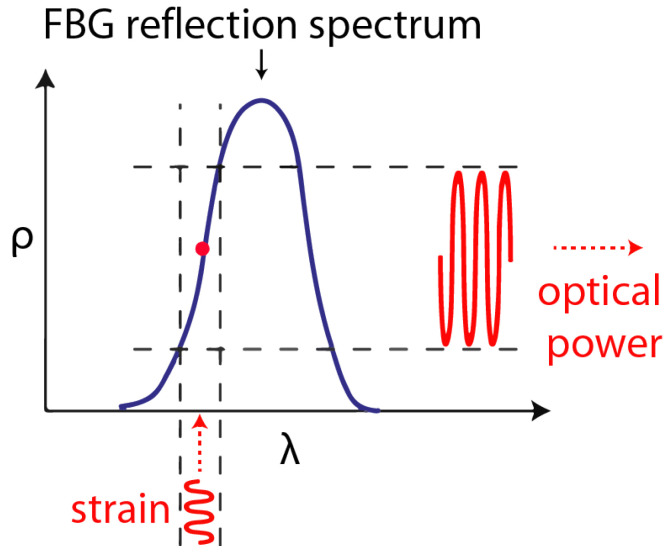
Increased sensitivity using the reflectivity slope.

Another configuration used to improve the sensitivity is the edge reflection configuration, in which a tunable or other narrow band laser source is set with its output wavelength on the linear region of the reflectivity spectrum of the FBG. A small shift in the $\lambda_{B}$ results in a large change in the output intensity, which can be detected by a photodetector, as shown in Figure 9.

The voltage signal (V) is proportional to the power of the incident light (P), grating slope of the FBG (G) and the change in $\lambda_{B}$ and is given by Equation (6) [65],

(6) $$V=\Delta \lambda_{B}GR_{D}P$$

where, $R_{D}$ is the photodetector’s response factor. The sensitivity can be improved by increasing the power of the laser, or using FBG with a higher slope such as the PSFBG. The limiting factor for the sensitivity is the SNR of the measurements. Kirkendall et al. [66] comprehensively discuss the sources of noise for high performance optical systems. Lissak et al. [65] experimentally identified the main sources for edge filtering applications as the laser power noise, the frequency noise and the shot noise. Wild et al. [67] differentially amplified the response of the edge filter system by using the transmitted and reflected signal together but did not address the systematic errors in the measurement system. Wu et al. [68] used a balanced photodetector and the PSFBG sensor to improve the SNR. The balanced photodetector measures the transmitted and reflected light simultaneously and can remove the laser power noise effects.

As mentioned before, interferometric sensors have one of the highest sensitivities among the FO sensors. FBG sensors interrogated with an interferometric approach can produce high sensitivity, while also allowing the benefits of the edge reflection approach. To form an interferometer, two FBG sensors are used to form a FPI. Multiple different approaches have been proposed in literature to interrogate the FPI. Pappu et al. [69,70] used a quadrature recombination technique. Two optical signals, set in quadrature to the most sensitive and the least sensitive region of the spectrum, are launched in the fiber and detected by the photodetector. Xu et al. [71] applied two FBG sensors with identical reflectivity separated by the desired cavity length. A change in the cavity length does not cause the central wavelength shift but increases the fringe density. The increased fringe intensity results in an increase in the power vs. wavelength shift slope, resulting in a higher sensitivity to the change in the structure. The challenge with this approach is the need for exact matching of the reflectivity spectrum, which is not possible using off-the-shelf FBG sensors. Furthermore, the change in the power is not linear, which makes signal processing a bit challenging. A similar approach using the cavity between FBG as a Mach-Zehnder interferometer was proposed by Koo et al. [72].

The above-mentioned demodulation approaches based on edge-filtering need calibration of the filter slope before they can be used. As a result, their application in in-service conditions remains a challenge. In addition, the maximum wavelength shift range is limited to the half of the FWHM of the sensor. For small amplitude GWs, this is sufficient, however in-service conditions often create a larger shift in the spectrum due to temperature or loading. For such applications, dynamic feedback control of the laser wavelength output must be included. As an alternative, Erbium doped fiber laser (EDFL) systems may also be used. The FBG and the EDFL are integrated, thus allowing auto-tunability in changing conditions. In this approach, the reflected light re-enters the laser ring cavity. The detected signal is a function of the ultrasound wave sensed at the FBG and the dynamic properties of the EDFL [73,74]. When the ultrasonic frequency is close to the relaxation oscillation frequency of the EDFL, an amplification is obtained, increasing the sensitivity of the system. This configuration has been used for multiplexing through cascaded ring cavities and FP filters [75]. The approach has shown excellent robustness to changing ambient conditions [76].

**Figure 10:**
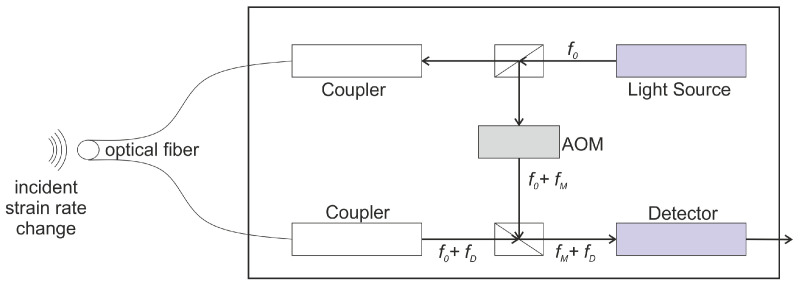
Optical circuit for FOD interrogation (Figure redrawn with permission from [79] ©Elsevier).

While FBG sensors are the most extensively used sensors, other sensors based on wavelength shifts, such as the sensor based on the Doppler shift (FOD sensor) and micro ring resonators (MRR), have been used for ultrasound detection. The FOD sensor is based on the laser Doppler effect. For a light wave of a particular frequency, traveling in a curved optical fiber, the frequency changes with the change in the strain rate of the fiber [77]. By monitoring the change in the frequency, the strain rate in the structure can be monitored. The change in the strain rate can then be correlated to the condition of the structure. The Doppler shift ($f_{D}$) is given by Equation (7) [78],

(7) $$f_{D}=-\frac{n}{\lambda_{0}}\cdot \frac{dL}{dt}$$

where, $\lambda_{0}$ is the wavelength in vacuum, $\lambda_{0}/n$ is the wavelength in the optical fiber, dL is change in the length in infinitesimal time dt. The Doppler shift can be measured using a laser Doppler velocimeter. The setup for the velocimeter is shown in Figure 10. For an incident light at frequency $f_{0}$ the frequency changes to $f_{0}+f_{D}$ due to the changes in the structure caused due the excitation. An acousto-optical modulator (AOM) changes the frequency from $f_{0}$ to $f_{0}+f_{M}$ in order to obtain beating signals with the desired frequency. By comparing the initial input frequency $f_{0}$ and the sensed frequency, the Doppler shift can be determined.

**Figure 11:**
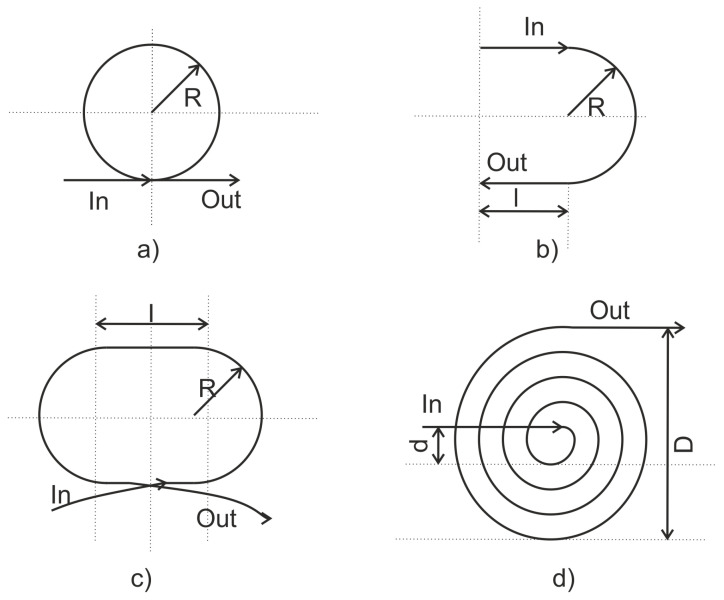
Different Configurations for FOD sensors (**a**) circle (**b**) U shaped (**c**) elongated circle (**d**) spiral.

Several different configurations of the sensor such as the circular, U shaped, elongated circle (ecircle), and spiral (Figure 11) have been proposed in the literature.

The sensitivity of the FOD sensor is proportional to length of the fiber and is given by Equation (8) [79].

(8a) $$\begin{array}{cc} f_{circle}^{th} & =-\frac{\pi Dn_{eq}}{2\cdot \lambda_{0}}\left(\epsilon_{x}+\epsilon_{y}\right) \end{array}$$

(8b) $$\begin{array}{cc} \;\;\;\;\;\;f_{Ushape}^{th} & =-\frac{n_{eq}}{\lambda_{0}}\left\{\frac{\pi D}{4}\left(\epsilon_{x}+\epsilon_{y}\right)+2l\epsilon_{x}\right\} \end{array}$$

(8c) $$\begin{array}{cc} \;\;\;\;f_{ecircle}^{th} & =-\frac{n_{eq}}{\lambda_{0}}\left\{\frac{\pi D}{2}\left(\epsilon_{x}+\epsilon_{y}\right)+2l\epsilon_{x}\right\} \end{array}$$

(8d) $$\begin{array}{cc} \;\;\;\;\;\;f_{spiral}^{th} & =-\frac{\pi \left(D^{2}-d^{2}\right)n_{eq}}{8\phi_{f}\lambda_{0}}\left(\epsilon_{x}+\epsilon_{y}\right) \end{array}$$

In the spiral and circular configuration, the sensor has isotropic sensitivity. Furthermore, as the sensor is composed by the simple optical fibers, it is cheap and easy to manufacture. The FOD sensor is found to be several times more sensitive than the FBG sensor in the wavelength division multiplexing configuration.

**Figure 12:**
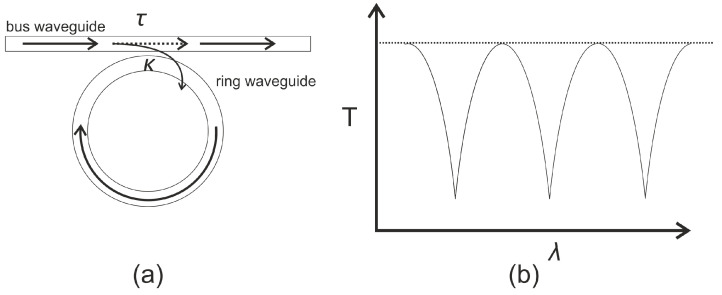
(**a**) schematic of MRR (**b**) transmission spectrum of MRR (based on reference [80]).

MRRs are similar to FBG sensors as they work as wavelength filters, the key difference being that the FBG produces a narrow bandwidth in the reflected spectrum while the MRR work produces the narrow bandwidth in the transmitted spectrum. The MRR consists of one waveguide (labeled bus) coupled with one or more loops of a ring waveguide. The schematic of the MRR is shown in Figure 12.

Resonance occurs when the round-trip phase acquired by the guided wave in the microring is equal to an integer multiple of 2π. Under such conditions, the optical wave returning to the coupler after a round trip is exactly π out of phase with the optical wave traveling through the coupler region in the straight waveguide. These two fields therefore interfere destructively, leading to a resonance dip in the transmission curve. The transmission coefficient for the MRR is given by Equation (9) [81],

(9a) $$\begin{array}{cc} \;\;\;\;\;\;\;\;T & =\alpha_{i}^{2}\frac{{\left(\tau -\alpha_{i}a\right)}^{2}+4\tau \alpha_{i}a\sin^{2}\frac{\phi }{2}}{{\left(1-\alpha_{i}a\right)}^{2}+4\tau \alpha_{i}a\sin^{2}\frac{\phi }{2}} \end{array}$$

(9b) $$\begin{array}{cc} \phi & =\frac{2\pi \eta_{eff}L}{\lambda } \end{array}$$

where $\alpha_{i}$ is the insertion loss, τ is the transmission coefficient, *a* is the attenuation factor, ϕ is the phase shift corresponding to a single pass inside the micro ring, $\eta_{eff}$ is the effective refractive index of the ring wave guide, and *L* is the circumference. For sensing applications, the incident waves affect the $\eta_{eff}$ due to the change in the cross-section of the waveguide as well as the elasto-optic effect [80]. The change in the $\eta_{eff}$ shifts the resonance frequency. This wavelength shift can be detected directly, or converted into an intensity change through the edge-filtering approach, similar to FBG sensors. The key benefits of the MRR is the small size of the sensor as compared to the FBG sensor. As a result, higher frequency waves can be more reliably sensed using the MRR. Unfortunately, for the wavelengths used, the micro-ring has to be very small, challenging the curvature limits of the optical fiber. The use of MRR for ultrasonic monitoring has been limited due to the difficulty in the fabrication of the devices. However, recent progresses in manufacturing technology including the nanoimprinting [82], lithography techniques [83] and 3D printing has made the manufacturing process cheaper and repeatable and the use of these devices is expected to gain more attention going forward [84,85].

While there are a large choice of optical fiber sensors for ultrasonic measurements, FBG sensors and their variants remain the most promising solution in the near-term. The next section describes recent advances in their use for GW sensing for SHM applications.

## 3. FBG Sensors for GW Sensing

Fiber Bragg gratings (FBGs) have been widely used for SHM in structures based on guided waves. As mentioned earlier, FBGs can provide high sensitivity to the low amplitude strain waves propagating through a structure, particularly when the slope of the FBG rising or leading edge is high. The key challenge to the measurement of GWs with any sensor is the need of a high signal-to-noise ratio (SNR), so that the waveform can be well resolved. The sensitivity of optical fiber sensors, including FBGs, is reduced by the small contact area between the fiber and the structure. This contact area can be significantly less than the comparable disc-shaped PZT sensors. In addition, the adhesive layer bonding the FBG to the structure typically demonstrates frequency dependent attenuation of the ultrasonic waves, with increasing attenuation at higher frequencies.

Four main approaches have recently been taken to increase the SNR for FBG sensors: (1) increasing the sensitivity of the FBG to a fixed amplitude guided wave; (2) focusing the guided waves in the structure prior to coupling to the FBG; (3) changing the fiber coating and coupling agent; and (4) remote bonding.

**Figure 13:**
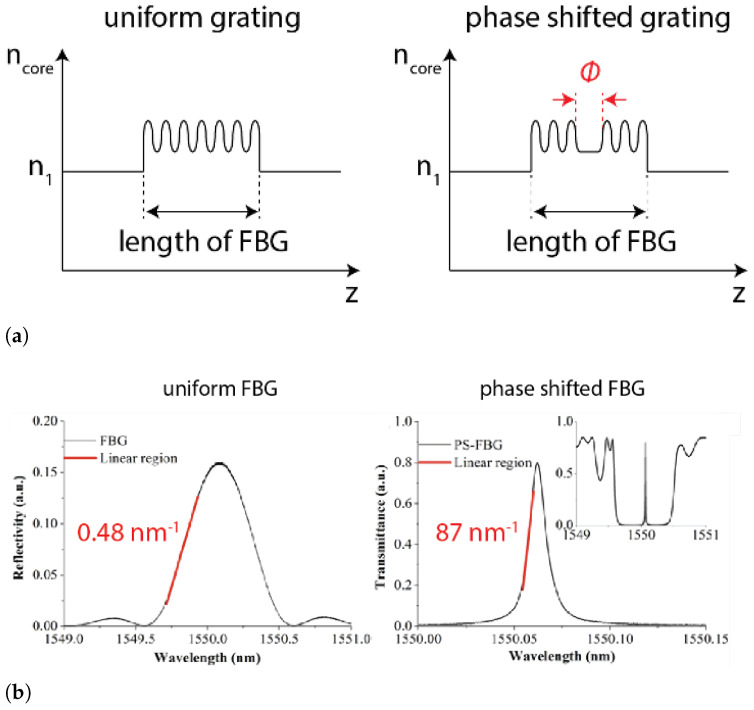
(**a**) Refractive index change of uniform and phase-shifted gratings and (**b**) the edge slope of the reflection spectrums for uniform and phase-shifted grating. Reprinted with permission from [68] ©The Optical Society.

The first approach is to maximize the sensitivity of the FBG sensor to the propagating wave in the fiber. As has been mentioned in the previous section, in the edge filtering configuration the sensitivity of the FBG is proportional to the slope of the FBG reflectivity spectrum. Thus, by modifying the index modulation of the FBG, this slope can be increased. For example, by keeping the other parameters the same, the slope can be adjusted by the length of the FBG. Long FBGs produce steeper spectral slopes, up to a limit [86,87]. Significantly higher spectral slopes have been achieved with phase-shifted FBGs (PS-FBGs). While producing the FBG, a π phase shift is introduced in the middle of the grating length as shown in Figure 13a. This shift results in an intermediate loss peak in the reflected spectrum with orders of magnitude and a higher slope than the leading or trailing edge of the standard reflected spectrum, as shown in the inset in Figure 13b. As a result of the increase in the slope, the sensitivity is improved [88]. The key drawback of the PS-FBG is the significantly increased complexity in the manufacturing of the FBG. Additionally, the width of the spectrum is drastically reduced, which leads to reduced robustness with changes in the ambient conditions. Active control feedback loops are often necessary to keep the input wavelength tuned to the narrow spectral peak. In addition to the sensitivity, the directionality of the sensor is impacted by the phase-shifted modulation. Liu and Han [88] showed that the phase-shifted length is much smaller than the ultrasonic wavelength, the sensor is essentially omnidirectional. However, as the FBG length increases, the sensor becomes more directional with the maximum sensitivity occurring at the normal incidence of the ultrasonic wave. Gatti et al. [89] combined the PS-FBG with a modulated laser source to reduce the laser power source noise and the frequency noise, further increasing the SNR. In this technique, a high frequency modulation (several hundred MHz) is applied to the laser. The ultrasonic signal received by the photodetector is then mixed with the electrical modulated signal and the error in the mixed signal is the ultrasonic signal.

Following the second approach of focusing the guided waves in the structure prior to the FBG, Sakai et al. proposed the use of an acoustic lens [90]. The lens amplifies the wave by acting as a wave guide. The refraction and resonant type waveguides indeed act as lenses and improve the signal amplitudes. Researchers have also bonded curved plate slices to the structure to serve as plano-convex lenses for Lamb wave focusing prior to the detector [91,92]. While all of these efforts have successfully increased the wave amplitude and therefore the sensor output, they result in the addition of a significant mass to the structure. As each FBG would require its own lens, this strategy is not suitable for multiplexed arrays of FBG sensors.

The third approach is to modify the optical fiber coating or coupling agent. Moccia et al. [93] used an intermediate coating between the FBG and the water to improve the coupling from the medium into the the fiber. While this soft coating well matched the impedance to the liquid environment, it is less suitable for the extraction of guided waves from solid structures. Takeda et al. [94] developed a small diameter optical fiber with 40 μm cladding and 52 μm polyimide coating to allow embedding in a carbon fiber composite and improving the amplitude of strain detected by the FBG. For the sensing of signals in the ultrasonic range, viscous coupling of the optical fiber to the structure provides an extremely high SNR, for example by using vacuum grease [95]. However, viscous coupling is not practical for many SHM applications where the sensor needs to remain connected to the structure. Lee and Tsuda [96,97] applied viscous coupling to create a ‘mobile sensor’ where the FBG sensor was bonded on an acrylic plate with a viscous couplant between the plate and the structure. The acrylic plate ensures more surface contact and as a result better coupling of the wave into the plate. Furthermore, the mobile sensor allows scanning of the entire structure rather than having the sensor at fixed locations. The mobile sensor was also insensitive to static strains due to the viscous coupling, thus allowing for better ultrasonic measurements.

**Figure 14:**
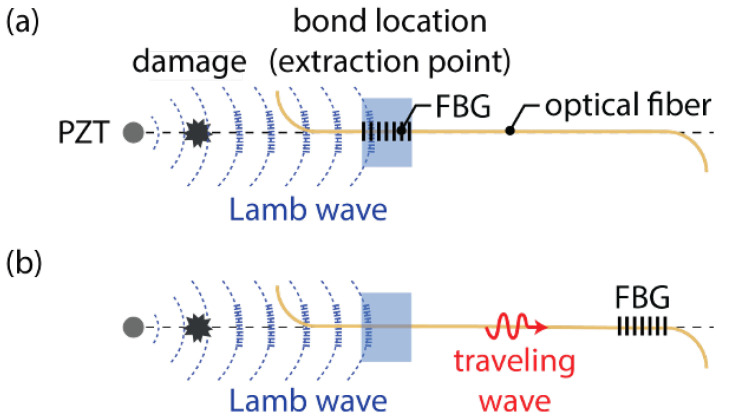
Ultrasonic signal measurement (**a**) at the extraction point (direct bonding) and (**b**) away from the extraction point using the FBG sensor (remote bonding).

The final strategy to increase the SNR for FBG detection of Lamb waves is referred to as remote bonding. In this configuration, the FBG is not directly adhered to the structure. Instead, the optical fiber is adhesively bonded to the structure at the location where the guided wave is to be extracted. The interaction between the adhesive bond and the structure creates guided ultrasonic waves, which propagate in the optical fiber. These propagating waves are also present when the FBG is directly bonded to the structure, but are not measured. For the case of remote bonding, shown in Figure 14, the FBG is located at a distance along the optical fiber, away from the bond, therefore the guided, ultrasonic wave propagating through the optical fiber is what is measured by the FBG. To better explain this behavior, the next sections describe the interaction between the propagating waves in the structure and the fiber as well as how the wave coupled in the fiber affects the FBG output response.

### 3.1. Remote Bonding of FBG Sensors

**Figure 15:**
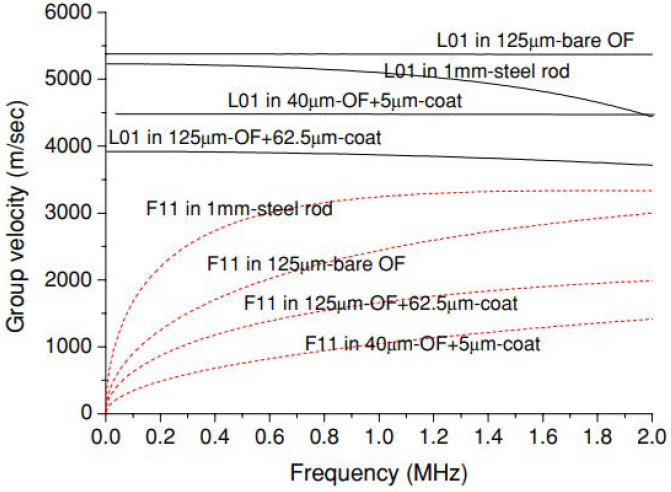
Dispersion curves for optical fiber of different diameters, with and without the polyimide coating. Dispersion curves of the reference steel rod are also plotted [98]. Reprinted with permission from [98] ©IOP Publishing.

For the case of remote bonding, the guided waves propagating in the structure (for example symmetric and anti-symmetric Lamb waves) are converted into guided acoustic modes in the optical fiber. It is these guided waves that are measured by the FBG or other optical fiber sensor. This conversion occurs where the optical fiber is connected to the structure, typically with an adhesive or adhesive tapes. Three potential guided ultrasonic modes exist for common optical fibers, longitudinal (L), flexural (F) and torsional (T) modes. The adhesive bond commonly suppresses the torsional mode but permits coupling of the longitudinal and flexural modes. Lee and Tsuda [98] calculated dispersion curves for the fundamental longitudinal (L${}_{01}$) and flexural (F${}_{11}$) modes in typical single-mode optical fibers and reduced diameter optical fibers. These curves are plotted in Figure 15. The fundamental longitudinal fiber mode ($L_{01}$) is non-dispersive in the sub-MHz frequency range for standard single-mode optical fibers [98,99]. In contrast, the flexural mode attenuates significantly faster than the longitudinal mode and is not detected by the FBG because it produces zero strain at the fiber core.

**Figure 16:**
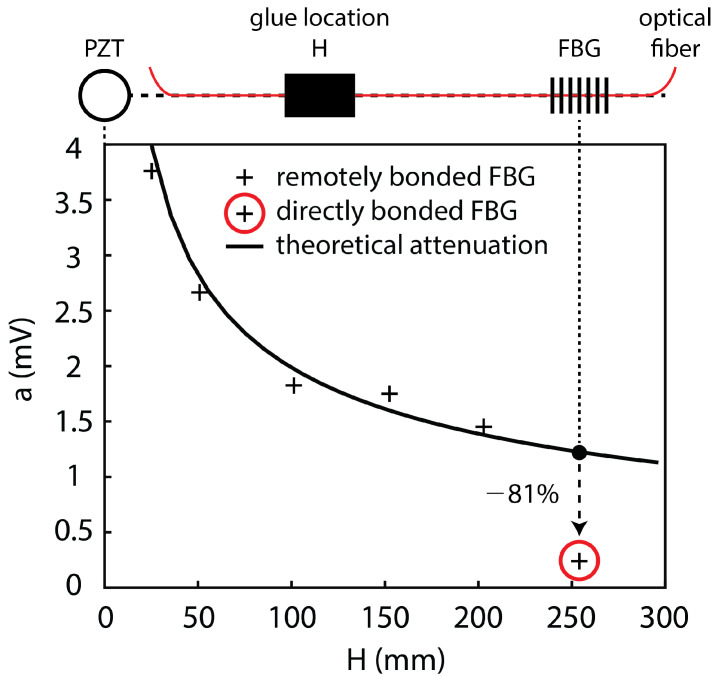
Theoretical amplitude of FBG output as a function of distance from PZT source and experimentally measured signal amplitudes. The circled data point indicates the directly bonded FBG [100].

Under certain conditions, using the remote bonding configuration can significantly increase the output signal from the FBG to the ultrasonic guided modes in the structure. For example, Wee et al. [100] demonstrated that directly bonding the FBG to an aluminum plate reduced the measured signal to 19% of the sensitivity of the remotely bonded FBG for the same configuration, as shown in Figure 16. Importantly, the waveform of the guided modes were preserved and both the S${}_{0}$ and A${}_{0}$ were successfully coupled into the L${}_{01}$ mode in the optical fiber. The fact that the coupled wave in the fiber can travel along the fiber long distances with minimal attenuation also makes the approach practical for sensor networks.

Several effects contribute to the increase in sensitivity for the remote bonding configuration, recently demonstrated through numerical simulations. Wee et al. [101] considered the effect of the shear lag through the adhesive bond. The shear lag effect is a function of the shear modulus and thickness of the adhesive. As the directly bonded FBG is in the adhesive region, the shear lag effect reduces the average amplitude of the wave transfer. Additionally, the remotely bonded FBG is measuring the $L_{01}$ mode, which can be enhanced through local resonance of the optical fiber-adhesive system [101]. Huang and Balusu [102] investigated this phenomena by treating the optical fiber-adhesive-structure system as an ultrasound coupler and calculating the scattering parameters for broadband time-frequency analysis. This analysis verified that the observed behaviors are due to ultrasonic resonances in the system and that the combination of material properties and geometries to produce the signal enhancement could be predicted numerically.

**Figure 17:**
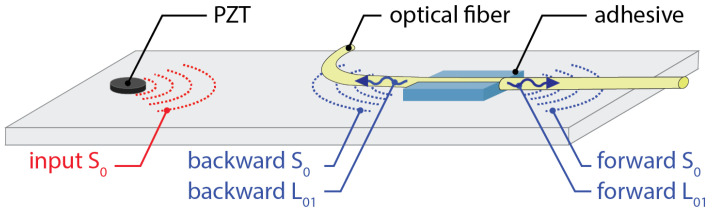
Coupling of $S_{0}$ to $L_{01}$ modes in optical fiber [103].

Apart from the potential increase in sensitivity to GWs, remotely bonded FBG sensors show several interesting phenomena which will be discussed in detail here. For example, propagating longitudinal modes are generated in the fiber in both directions from the bond, as shown in Figure 17, not just in the direction of the original Lamb wave. The directions are labeled as forwards (in the same direction as the original Lamb wave) and backwards (the reverse direction) in Figure 17. The relative amplitudes of the forwards- and backwards-coupled waves in the fiber depend on the type of bond used, the length of the bond as well as the properties of the adhesive [103]. Further, if the $L_{01}$ wave encounters another bond further along the fiber, a portion of the wave is reflected back into a backward propagating $L_{01}$ mode and a small portion of the wave is coupled back into the structure. This interaction of the mode with other bond locations may introduce errors into the signals from multiplexed sensors or complicate the signal processing. To avoid this, proper management of the fibers and multiplexed sensor array is necessary.

**Figure 18:**
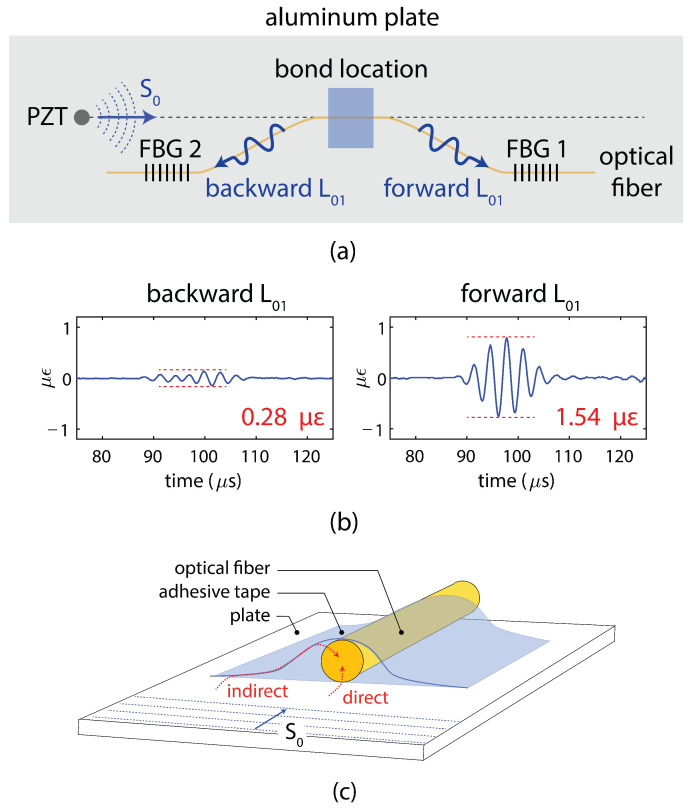
(**a**) Experiment and (**b**) measured forward and backward coupled L${}_{01}$ modes for S${}_{0}$ mode coupling to forward and backward L${}_{01}$ modes for remotely bonded FBG. The bond is adhesive tape. Strain peak-to-peak amplitude is also given in each plot. (**c**) Schematic of indirect and direct ultrasonic pathways [104].

The directionality of the coupling from ultrasonic waves into the optical fiber can also be tuned through the choice of different bonding methods. For example, Wee et al. [104] demonstrated tuning of the two directions using a standard adhesive tape to bond the optical fiber to an aluminum plate. Figure 18a,b shows an experiment and measured signals for this case. The amplitude of the forward coupled L${}_{01}$ mode is approximately five times that of the backward coupled mode. The exact ratio between them can be tuned by varying the tension in the tape. The source of this directional difference is in the fact that there are two pathways by which the ultrasonic signal is coupled to the optical fiber, as shown in Figure 18c. The first pathway, labeled direct, is through direct contact between the optical fiber and the aluminum plate surface. The second pathway, labeled indirect, is through coupling of the ultrasonic wave into the adhesive tape, propagation along the tape, and coupling to the optical fiber at the top interface. This pathway produced negligible coupling to the backwards L${}_{01}$ mode in the optical fiber. The relative amplitude of the coupled signal through the each path depends on the tension applied through the adhesive tape and the material properties.

### 3.2. FBG Length Effects

As with any ultrasonic sensor, the relative gauge length of the sensor compared to the wavelength of the ultrasonic wave to be measured is a critical factor in the sensor output. Betz et. al. [105] first noted that the reflected spectrum of a FBG when receiving the Lamb wave is a complex function of the resulting strain, because the strain due to the ultrasonic wave is non-uniform along the length of the FBG.

Generally, the sensor output is dependent on the ratio of the wavelength of the propagating wave ($\lambda_{GW}$) to the gauge length (*L*) of the FBG. The earliest work by Coppola [106] and Minardo [107] identified three regimes based on the $\lambda_{GW}/L$ ratio. In the regime where $\lambda_{GW}/L\gg 1$, the grating undergoes uniform deformation, leading to a shift of the Bragg wavelength proportional to amplitude of the GW. Takeda et al. [94] , Betz et al. [105] Culshaw et al. [108], Thursby et al. [109], all recommended that for a sufficient Bragg wavelength shift, the relative ratio should be in this regime. The recommended ratio for the reliable shift in Bragg wavelength was determined between six and seven by Takeda et al. [94] and Culshaw et al. [108]. Betz et. al. [105] demonstrated that in this range, the linear assumption produces an accurate reconstruction of the symmetric or anti-symmetric Lamb waves. This limit on the length of the FBG also limits the size of damage that can be detected with a FBG sensor network. Although the excitation frequency of the input Lamb waves could be changed, the condition that $\lambda_{GW}$ is smaller than the defect dimensions must still be met.

However, it is not always possible to select a FBG length to satisfy the condition of $L_{FBG}\ll \lambda_{GW}$ , particularly for higher frequency Lamb waves in the MHz range. In this range, Lamb wavelengths in thin metallic structures are on the order of millimeters. In the transition regime where $\lambda_{GW}/FBG_{L}≊1$, the Bragg wave length undergoes a shift, but also the bandwidth of reflection spectrum increases with decreasing *L*. In this region, the sensitivity increases approximately linearly with $\lambda_{US}/L$. Some recent work by Goosens et al. [110] has given insight into the response of the FBG spectrum to the GW in the regime II. The result investigates waves at 50 kHz and 250 kHz with sensors from five different gauge lengths, namely 2, 3, 5, 8, and 10 mm. The ratios achieved are in regimes I and II. By sweeping the wavelength over the entire reflectivity range of the FBG sensors, the FBG spectrum has been reconstructed. They show that for regime I the Bragg wavelength shift is seen for a while, and for regime II the widening of the peak is seen. The widening of the peak is due to the non-uniform strains on the different parts of the FBG. The coupling mechanisms, namely Bragg wavelength shift ($\Delta \lambda_{B}$)and the peak widening (Δ FWHM) for the different ratios, were given and their contribution to the GW measurement is determined. The study shows that the FBG sensors can be reliably used for GW sensing even in regime II, albeit the main mechanism of transduction is Δ FWHM rather than the peak shift. This paves the way for the use of FBG sensors with longer gauge lengths, which improve the sensitivity without losing out on the ability of the FBG sensors to detect higher frequencies of GW.

In the third regime, where the $\lambda_{GW}/FBG_{L}\ll 1$, the original index modulation of the FBG as written and the index modulation of the FBG due to the wave induced strain are of the same order, creating a beating interaction between the two. This behavior results in a damped oscillatory behavior for $\lambda_{GW}/L<$ 0.8. Generally, the sensitivity of the FBG is low in this region.

To solve the problem of achieving a sufficiently high $\lambda_{US}/L$ ratio at higher Lamb wave frequencies, Davis et. al. [111] developed a specialized fabrication process for short FBG sensors. Through a combination of specialty photosensitive optical fibers and a variable aperture lens in the frequency doubled AR-ion laser beam path during writing of the FBG, the authors were able to write FBGs with a 75% (6 dB) reflectivity at lengths down to 200 μm. The authors defined a stricter criterion for successful measurements than Minardo et al. [107] and Betz et. al. [105], i.e., the measured wave should have 98% correlation with the input Lamb waves, $\lambda_{GW}/L\geq 8.8$. Even with this criterion, Davis et. al. [111] were able to measure Lamb waves in a 0.8 mm thick aluminum plate at wavelengths down to 2 mm, corresponding to 1.1 MHz for the A${}_{0}$ mode and 2.4 MHz for the S${}_{0}$ mode.

### 3.3. Directionality

**Figure 19:**
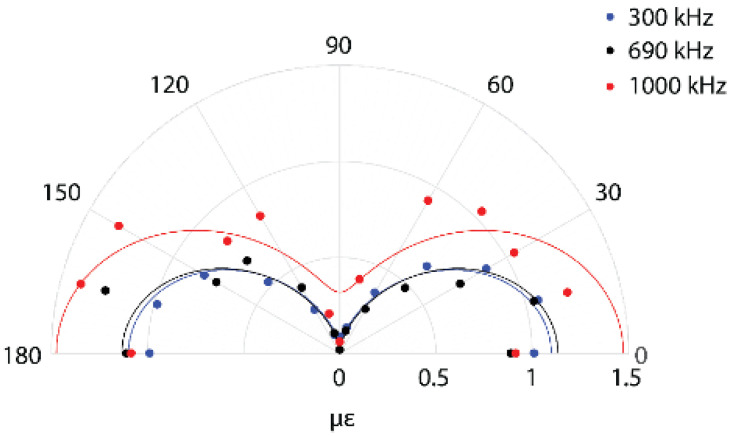
The angular response of directly bonded FBGs to S${}_{0}$ mode with different excitation frequencies plotted in polar coordinate system.

For an isotropic structure, when a strain sensor has displacement continuity with the structure surface (i.e., it is perfectly bonded to the structure), the sensitivity of the strain sensor with respect to the principle axis follows,

(10) $$\epsilon_{axial}=\epsilon_{1}\cos^{2}\theta +\epsilon_{2}\sin^{2}\theta$$

where, $\epsilon_{axial}$ is the strain along the sensor axis, $\epsilon_{1}$ and $\epsilon_{2}$ are the principle strains and α is the angle with respect to the direction of $\epsilon_{1}$ [112]. For far field measurements, the propagating wave front can be assumed to be planar, and the second term reduces to 0. As a result, the axial strain in the FBG is proportional to $\cos^{2}\theta$. Several researchers have experimentally investigated this, and confirmed the analytical expectations [94,113]. Example data for a FBG sensitivity to a $S_{0}$ Lamb wave at three different frequencies is shown in Figure 19. According to the analytical prediction, the sensor should show 0 value for a perpendicular wave, and experiments should show a finite value, albeit low, for the perpendicular wave. This may be attributed to the distortion of the FBG due to the transverse load, leading to the widening of the peak resulting in some detectability of the perpendicular wave and the finite length of the FBG. A similar directionality was observed for the PS-FBG sensor in the far-field region, but it is more complex for a shorter distance of propagation [114].

**Figure 20:**
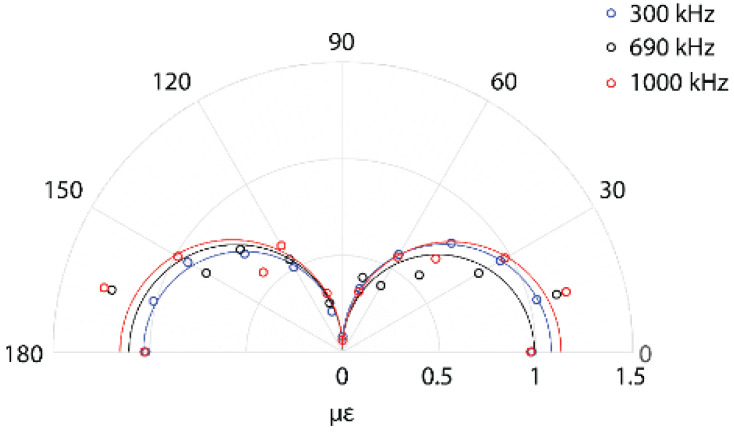
The angular response of remotely bonded FBGs to S${}_{0}$ mode with different excitation frequencies plotted in a polar coordinate system.

For the remote bonded configuration, experimental studies reveal a cosθ proportionality as opposed to the $\cos^{2}\theta$ proportionality for the direct bond [113], as seen in Figure 20. This is the same behavior as observed for sensors coupled through viscous bonding, where the transfer is through force rather than displacement [115]. Based on these observations, it can be concluded that the coupling of the Lamb wave to the acoustic mode in the optical fiber follows the force continuity principle rather than the displacement continuity.

Due to the directionality of FBG sensors, they have been considered by some researchers as inappropriate for GW measurements. In order to overcome this directionality, Giurgiutiu et al. [116,117] developed a piezo-optical ring based sensor. The FBG sensor is mounted on a metallic ring structure. The propagating wave in the structure gets coupled to the ring and goes into resonance [116,117]. The resonant frequency of the resonance can be tailored by modifying the geometrical properties. The ring structure allows omni-directional sensing and sensitivity to out of plane vibrations, and, due to the resonance, an amplification of the GW is achieved. Unfortunately, due to the metallic ring structure, the construction is bulky and compromises the benefits of the use of FBG sensors. Furthermore, as the resonance frequency is fixed, the versatility of the setup is limited.

### 3.4. Detection of Multiple Modes

Much of the SHM applications using guided waves actuated in a thin structure using PZTs or other actuators have focused on frequencies at which only the fundamental guided modes, A${}_{0}$ and S${}_{0}$, are possible in the structure. The main motivation is to reduce the number of propagating modes and therefore the complexity of the damage identification problem. When only the fundamental modes are present it is often possible to separate the waves in time at any sensor location, due to their different propagation velocities in the structure. In contrast, when more modes are present, this time separation may not be physically possible at all sensor locations, particularly when multiple back reflections are present. In addition, by choosing a mode that is relatively non-dispersive, for example the $S_{0}$ mode, measurements do not need to be made with a high spatial density in the structure [118].

**Figure 21:**
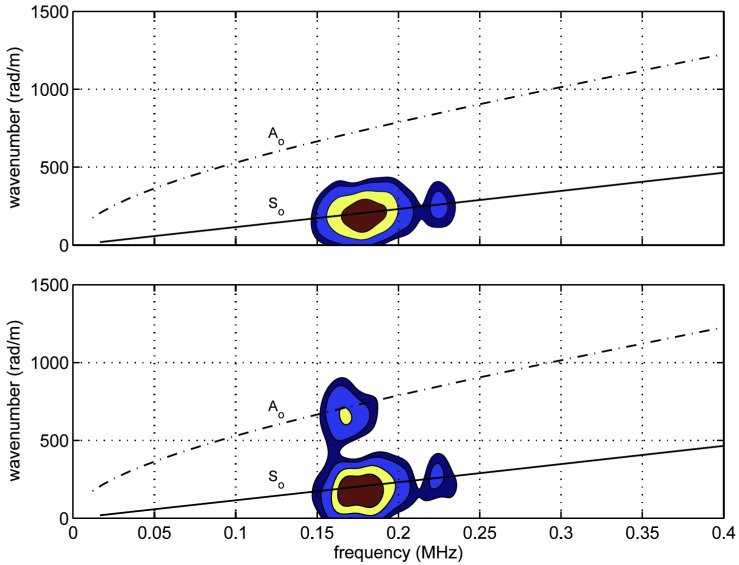
Spectral decomposition of a FBG sensor array response measured for a selectively excited S${}_{0}$ mode in the absence of the bonded disc (**top**) and after attachment of the disc (**bottom**) [118]. Theoretical dispersion curves are also shown. Reprinted with permission from [118] ©IOP Publishing.

On the other hand, collecting signals from multiple high order modes, however, can provide high fidelity information about the state of the structure, since these modes have much stronger interactions with flaws in the structure [119]. Rajic et. al. [118] exploited the multiplexing capabilities for a large numbers of FBG sensors in a single optical fiber to create a spatially and temporally distributed sensor array, which can then separate high order modes through 2D FFT signal processing. The wavelength of the Lamb waves that can be resolved by this technique is bounded by the limits,

(11) $$L\ll \lambda_{GW}\leq \left(N_{g}-1\right)\Delta x$$

where $N_{G}$ is the number of Bragg gratings and Δx is the center-to-center separation between them. Rajic et al. [118] experimentally demonstrated the technique on a thin metallic plate using 14 FBGs of length L = 1 mm and center spacing Δx = 1.5 mm. Despite the fact that the FBGs were relatively short, they were high reflectivity FBGs with a maximum reflectivity ≥95% and a bandwidth of approximately 0.8 nm (see Davis et al. [111] for the grating writing process). A $S_{0}$ mode was launched in the plate. Figure 21 shows the 2D fast Fourier transform (FFT) processed data from the FBG array for two cases: the original plate and after a disk is bonded to the plate to simulate damage. In the second case, partial mode conversion from the original $S_{0}$ mode to a $A_{0}$ mode can be seen clearly once the disk is added to the plate. The data in Figure 21 were obtained without taking into account the time of arrival difference between the two modes.

**Figure 22:**
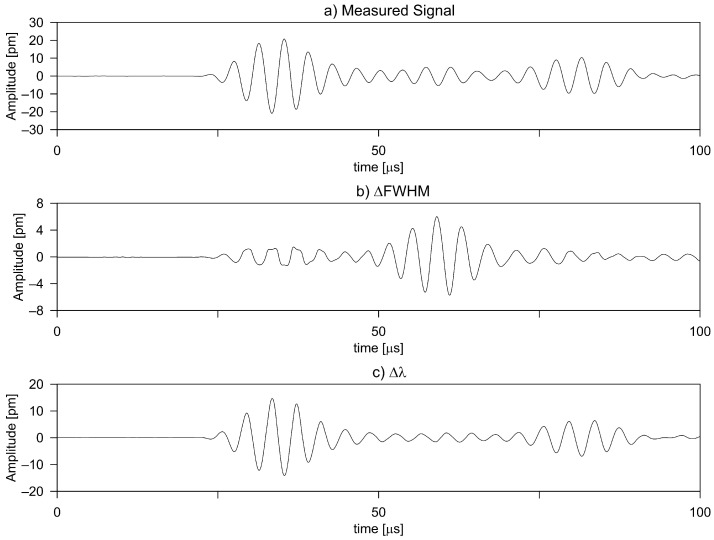
Comparison of time signals for the raw data and the modulation in FWHM and $\lambda_{B}$ for 250 kHz [120].

Furthermore, the fact that the sensitivity of the FBG to different wavelengths is different in the $\lambda_{GW}/FBG_{L}≊1$ regime (see Section 3.2) can be used to develop a mode separation technique for FBG sensors. Figure 22 shows the results for the excitation of 250 kHz wave in a simple aluminum plate with a PZT actuator at 0.125 m from the FBG sensor. The sweep of the spectrum of the FBG was obtained similarly to Goossens et al. [110] and the mechanisms for the coupling of the A and S mode were determined. For the 1 mm aluminum plate, the wavelength of the A${}_{0}$ wave is 5.6 mm while that of the S${}_{0}$ wave is 20 mm. Using the FBG with a 10 mm gauge length, the two modes have different $\lambda_{GW}/FBG_{L}$ ratios and as a result have different mechanisms for affecting the FBG spectrum. This mode separation may simplify the signal processing of the GW and can readily find applications in SHM.

## 4. Optical Fiber-Based Ultrasonic SHM

A recent review by Wu et al. [16] covers much of the literature on FBG sensors for GW detection prior to 2018. So, the primary focus of this paper is on fiber optic sensors other than FBG sensors for GW detection, and more recent advances in FBGs for GW detection. Similar to Wu et al. [16] we classify SHM processes into impact detection, acousto-ultrasonic (using actuators and sensors) or acoustic emission. The distinction is made typically on the source of the ultrasonic signal, the amount of energy in the system, and the dominant frequency range.

### 4.1. SHM Using Sensors Other Than FBG Sensors

As mentioned in earlier sections, there are several different types of transduction techniques. The earliest work in acoustic emission detection was using the intensity based microbend sensor for composites [121,122]. A laminated composite was subjected to tensile tests, and when cracking occurs, the micro-bend sensor detects the acoustic event. The setup needed for the acquisition is simple, but the signal processing requirements were deemed high according to the state-of-the-art in algorithms 20 years ago. The main drawback is the high noise content, which makes the signal processing and extraction of damage sensitive features a challenge.

The sensors based on interferometric principles are known to be extremely sensitive to ultrasonic waves and hence have been commonly used. The work by Liang et al. [123] made use of a Mach-Zehnder interferometer. In their work, the acoustic emission event may be detected by measuring the time delay between the signals of two MZIs. The setup has been validated for remote sensing, with a range of up to 20 km. The localization accuracy with this setup is of the order of 1%, which is desirable for distributed sensing. Xu [124] also make use of a MZI for detecting delamination in composite structures. The sensor configuration makes use of the MZI with a 2 × 2 coupler and a 3 × 3 coupler in order to convert the change in intensity of the light to a change in the phase to obtain the interference pattern. The MZI has been implemented for notch detection in pipes by Zhou et al. [125]. All these techniques show the readiness of the MZI-based sensors for SHM applications. Due to the transduction principle, they may be used for distributed measurements, which are essential in large civil engineering structures. The key problem with these MZI-based techniques is the relatively lower AE frequencies that may be captured. To overcome this, a double MZI approach was proposed by Kong et al. [126]. The proposed approach improves the localization accuracy even further while enhancing the frequency range of the signal acquisition. The interferometric principle has also been used by Fracarolli et al. [127] for monitoring of bushings in power transformers. The acoustic event makes changes in the frequency of the light propagating in the sensing arm, which is coupled with the light with a carrier frequency. The resulting beating consists of the two components, which can be captured. The optical fiber sensors are ideal in this application due to the presence of a large voltage in the transformer. A similar concept of the beating frequency has been used for SHM using Doppler effect-based sensors. The FOD sensors have been successfully applied for debonding detection in lap joints [128] and corrosion detection [129].

In addition to the interferometric sensors, polarization-based sensors have been applied for AE detection. Pitropakis et al. [130] applied them for impact detection composite plates, while Verstrynge et al. [131] used polarization-based sensors for AE detection in arches. They validated the sensor results in three point bending tests on actual masonary structures. The polarimetric sensors were also used by Thursby et al. [132] for the detection of a hole in the plate. The authors commented that the polarization-based sensors are distributed sensors, which do not need expensive equipment for monitoring. However, the directional nature of the sensor and the distributed measurements may make signal processing challenging [133]. Rapid developments in optical technology, such as tunable lasers, have since made other FO sensor interrogators less expensive. Therefore, these benefits may be less critical.

An upcoming trend for AE sensing is the use of the fused micro-fiber coupler. It has been successfully used as an AE sensor and for impact localization [134]. The extremely small size of the coupler is seen as an advantage of the sensor and makes embedding of the sensor in a material system feasible. The interrogation setup necessary is not resource intensive and the signal processing requirements are low. Furthermore, very little calibration is necessary, all of which makes the micro-fiber ideal for in-service condition monitoring. A fused coupler has been used for AE-based damage detection in wind turbine blades [135] and has the potential to be applied for other applications [136]. Fu et al. [137] developed a neural network for source localization based on the micro-coupler sensor.

Several commercial systems making use of scattering-based sensors have been proposed. Bao et al. [138] provide an excellent overview of the use of the sensors for monitoring different parameters, such as vibration displacement as well as acoustic emission. Qin et al. [139] increased the maximum detectable frequency using time division multiplexing with the OTDR system. A two-pulse system was developed, where the short pulse allows for good spatial resolution while the longer pulse allows for the detection of ultrasonic frequencies. Recently, Agarwal et al. [140] presented the use OTDR for crack detection in water pipes. They showed that the acoustic wave frequency is a function of the size of the crack, and large cracks may be detected with a OTDR setup capable of capturing 100 kHz frequency.

### 4.2. SHM Using FBG Sensors

FBG-based techniques indeed are the most popular among the FO sensor technologies for GW-based SHM. Since the review paper by Wu et al. [16] in 2018, more papers have been published for GW-based SHM using FBG sensors than all other optical fiber sensors combined. Therefore, we divide this section into the different SHM processes using FBG sensors.

#### 4.2.1. Impact Localization

For impact localization, FBG sensor-based detection has typically been based either on standard WDM interrogators (low sensitivity) or the amplitude encoded edge filtering methods (high sensitivity), as discussed previously. The novelty of the work in this area is therefore mostly through the development of novel algorithms for improved localization.

Shrestha et al. [141] used the WDM approach with a fast interrogator. They developed the error outlier technique for the impact localization on a composite wing structure. A training set was prepared with known impact locations and the FBG response was recorded. At a later time, when a test signal is recorded, the test signal is compared with the training set and the outliers are determined for all sensors, which are then used for impact localization. In the method proposed, the use of the subset of sensors, selected based on the outlier properties, improves the impact localization considerably. In the same research group, Jang et al. [142] replaced the outlier technique with an artificial neural network for the localization of impact in a stiffened composite plate, with similar results. Yaozhang et al. [143] similarly used artificial intelligence for impact localization using FBG sensors. Jang et al. [144] further expanded the work by incorporating not only the signal strength but also the time of arrival for improving the impact localization. By incorporating the time of arrival, the effect of the directionality of the sensors was somewhat minimized.

Wang et al. [145] used variational mode decomposition (VMD) to enhance the signal strength. The higher signal strength, along with the use of a Teager energy operator, allows for a more precise determination of the time of arrival of the wave and in turn localization of the impact. A slight modification of the VMD approach is the use of the empirical mode decomposition (EMD). Lu et al. [146] applied EMD for impact localization in composite plate with varying thickness. The magnitude of impact for the varying thickness was adjusted through the use of an energy feature set. The energy feature set is identified based on the cross-correlation coefficients of the raw signal and the decomposed modes of the EMD. The results indicate that indeed through the use of EMD-based features the impact location can be accurately detected. The cross correlation was also used by Vorathin et al. [147] who developed cross correlation-based linear source localization algorithm for real time impact localization.

Chen et al. [148] presented that the key limitation of FBG sensors for impact localization stems from their directional sensitivity. They show the limited effectiveness of a single FBG sensor for impact localization. They proposed the use of two orthogonally placed FBG sensors. By appropriate choice of the Bragg frequency of the gratings and the sensor rosette, the directionality effect was overcome and the impact localization improved. The time difference of arrival was used for the localization.

The impact detection in a honeycomb structure was studied by Majewska et al. [149]. Five sensors at 15 degree angle intervals were used to overcome the directional sensitivity of the FBGs. Hammer and steel ball drop impacts were introduced. A standard WDM interrogator was used, which results in a much lower sensitivity of the FBG output to the waves. As a result, the impact localization was imprecise. Also, the complex honeycomb structure and the damping made the signal processing a challenge. In an extension of this work, the authors used embedded sensors for impact detection in a sandwich panel [150], with similar results.

#### 4.2.2. Acoustic Emission

Acoustic emission signals are typically low energy and high frequency broadband signals that are generated during crack formation or damage propagation. Several passive systems for their detection have been proposed, making use of FBG sensors. Some of the key publications have been reviewed by Willberry et al. [33]. More recently, Jinachandran et al. [151] performed a variety of experiments for AE-based detection in weld structures. They first proposed a novel packaged FBG sensor that is robust and applied it in conditions simulating in-service conditions due to hydrogen induced cold crack monitoring. Xu et al. [152] employed FBG sensors for debond monitoring of T-joints using cantilevered FBG sensors. The T-joint is a very important structural component of aerospace structures and the FBG sensors offer an inexpensive method for the assessment of the debond.

Violakis et al. [153] studied the effect of the diameter of the optical fiber on the sensitivity of the FBG as AE sensors. They concluded that the use of smaller diameter FBG are more suitable for AE detection as their sensitivity is higher over a larger frequency range.

Recently, advances have also been made in the signal processing of the FBG sensors for acoustic emission events. Pang et al. [154] developed a barycentric coordinate-based algorithm for acoustic source localization. They used the Morlet wavelet transform to enhance the features and simplify the identification of the velocity and time of arrival. Fu et al. [155] trained an artificial neural network to analyze the complex non-linear relationship between the AE wave source location and the arrival time. They analyzed a 3-dimensional polymer bonded explosive structure. The neural network has its limitations as the quality of the training data severely affects the localization accuracy, but through proper training and large data sets it has the potential to be useful for AE source localization.

Almost all methods for AE source localization make use of triangulation with three or more sensors. An innovative approach was proposed by Yu et al. [156] who used a single FBG sensor for the localization of the acoustic event. The continuous wavelet transform allows the separation of the dominant frequency and the arrivals of the two modes (anti-symmetric and symmetric). The difference in the velocity of the two modes is used to determine the location. They made use of the remotely bonded configuration and showed that the output sensitivity is independent of the distance between the bond and the FBG sensor.

#### 4.2.3. Acousto-Ultrasonic

For acousto-ultrasonic detection systems, piezo-electric actuators are typically used for generating GWs in the structure. These GW are then detected using FBG sensors. These systems are often referred to as hybrid systems, as they make use of two different sensor technologies. The key issues to tackle for such an hybrid system is ensuring the multiplexing ability of the sensors and tackling the challenges associated with the directionality of the FBG sensors. The directionality challenges can be overcome through innovative signal processing techniques.

Boffa et al. [157] developed such an hybrid system, which makes use of surface-bonded as well as embedded FBG sensors in a composite structure. They used an off-the-shelf optical fiber sensing interrogator from Redondo optics (FAESense M400), which uses two-wave mixing integrated microchip technology. The authors showed that multiplexing and tuning of the frequencies is possible along with a very high sampling frequency. The results clearly show that the packaged system captures the GW signal with very low distortion and can be implemented on any smart structure.

Lambinet et al. [158] developed a hybrid system for detecting barely visible damage in a patch-repaired composite. The method outlined provides temperature compensation by taking into consideration the changes in the GW propagation due to the temperature as well as the effect of temperature on the FBG spectrum. The method successfully detects damage at higher frequencies, owing to the higher contribution of the S${}_{0}$ mode. The temperature compensation tends to limit the contribution of the A${}_{0}$ mode and is given as the reason for the selective sensitivity.

Soman et al. [159] propose a two-step damage detection process. The two-step method not only overcomes the passive nature of the FBG sensors, which reduces the number of actuator-sensor pairs, but also overcomes the directional sensitivity of the FBG sensor. In the first step, the damage is detected and the hot spots identified based on the time of arrival of reflections from the damage location. For identifying the exact location, edge reflections are used. The edge points of reflections act as pseudo actuators, allowing for more actuator-sensor paths, thus overcoming the passive nature. By taking the ratio of the relative amplitude for a particular path, the directional sensitivity is accounted for. The work was extended with an hyperbola-based approach, and shows that the ellipse -based approach gives a closed form damage localization, thus improving the damage localization [160]. The time difference-based approach is more suitable in the remote bonding configuration, where there is uncertainty in the location of the wave coupling or the velocity of the wave propagation in the fiber. This is particularly useful in applications with embedded FBG sensors.

Sun et al. [161] proposed the use of a phased array of FBG sensors. They used the delay-and-sum beam forming principle to overcome the directional nature. The experiments on a simple aluminum plate show that indeed damage can be located using this technique. The method was then expanded with an adaptive minimum variance method to improve the damage detection [162]. These works successfully overcome the directional sensitivity through the use of multiple FBG sensors in a particular configuration.

Another innovative configuration solution to overcome the directionality limitations was proposed by Yu et. al. [163]. They used a remotely bonded PS-FBG with two orthogonal bonds, which was able to diminish the directional coupling of the waves. Furthermore, they developed a technique for visualization of the wave propagation along the structure using non-contact excitation and a single remotely bonded structure. For a linear structure, the visualization is equivalent to a single-excitation and multi-point measurements, which are undertaken by a laser Doppler vibrometer. The full wave field data is information-rich and may be used with advanced signal processing techniques for the detection of damage. Another work making use of the remote bonded configuration was used for the monitoring of a structure at very high temperatures (1000 °C) [164]. Through the full wave field data, the mode components were separated using 3D fast Fourier transform (FFT) and used for damage visualization. Wu et al. [165] used the non-linear ultrasonics for detecting fatigue cracks. The PS-FBG was used for the detection and the performance was compared with FBG sensors and PZT sensors. The performance of the PS-FBG was found to be consistently better over a large frequency range.

**Figure 23:**
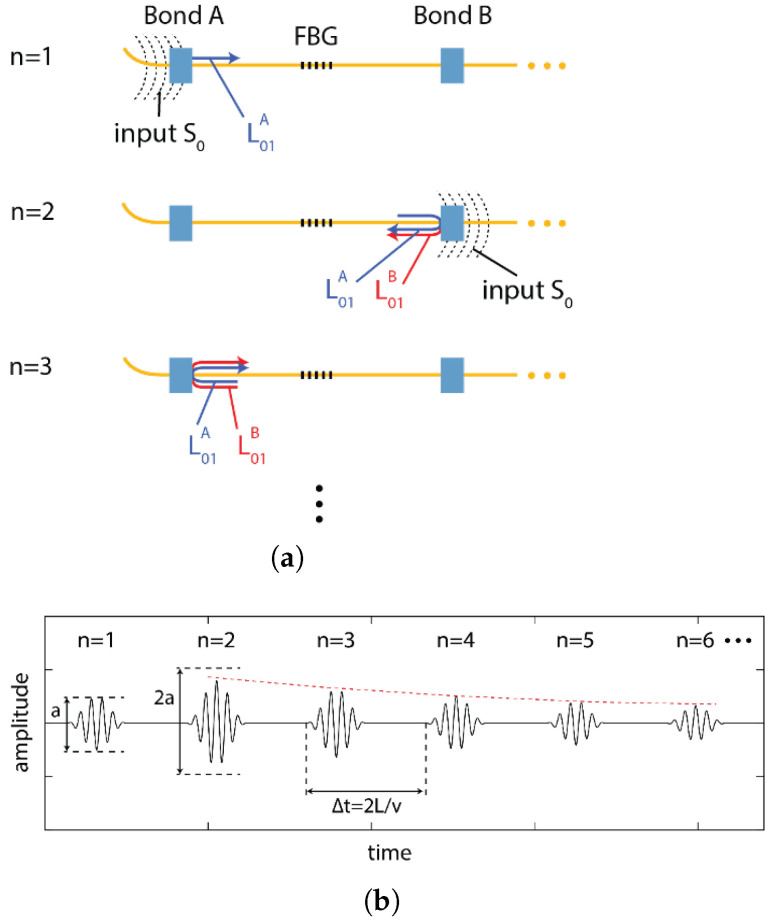
Concept for self-referencing ultrasound sensor. (**a**) Pathways for first three arriving wave packets at FBG (n = 1, 2, 3, …) and (**b**) corresponding theoretical output FBG responses with the wave number, n. [166].

The remotely bonded FBG sensors were used for reference free damage detection by Wee et al. [166]. They proposed a setup, as shown in Figure 23, where the FBG was remotely bonded on either side. The wave propagating will carry differential information, as the coupled wave at bond 1 will carry information without the damage, while the wave coupled at bond 2 will carry the damage information. This reference-free technique can overcome ambient condition changes, although it is limited only to damage detection.

As mentioned in the previous section, the interferometry-based sensors are more sensitive than the amplitude modulated sensors. In the approach by Xu et al. [71] two FBG sensors with the same wavelength were used as mirrors in the FPI. The strain in the structure coupled to the fiber does not change the spectrum but changes the fringe density, which is used for detecting the ultrasonic wave. The higher SNR achieved by the setup allows for better damage detection. A similar concept has been employed by Ismail et al. [167]. The additional wave packet due to the damage, the delay in the time of arrival, and change in the amplitude were used for detecting a slot type damage in an isotropic structure.

All the above techniques make use of additional actuators for the damage detection. The need for additional actuators can undermine the benefits offered by the FBG sensors. In order to overcome this, Druet et al. [168] and Recoquillay et al. [169] developed a technique which is based on the passive acquisition of the signals. The passive signals from ambient excitation are deconvolved on the frequency range of interest. The EXCITELET tool is used for the localization of a simulated damage in a CFRP plate.

Finally, based on the above discussion, several optical sensors may be used for each of the goals of impact detection, acoustic emission-based SHM and acousto-ultrasonic SHM. The different sensors all have different advantages and limitations, so we summarized these below in Table 1.

## 5. Conclusions and Future Trends

The industry standard for GW- and AE-based SHM are piezo-electric-based systems because of their lower cost and wider commercial availability. However, optical fiber systems provide key attributes, such as immunity to electro-magnetic interference and their ability to be easily multiplexed. Large, dense networks of sensors can be easily installed and interrogated for structural systems. The gauge length and resolutions of the measurements can also be tuned based on the choice of optical sensor applied. Although the cost of some OF sensors and interrogators can be much higher than equivalent piezo-electric transducers, the fact that a single interrogator can monitor a large number sensors means that the cost of the overall SHM system can be lower.

One challenge to optical fiber systems for GW measurements is that edge filtering interrogation techniques are required to measure the high frequency, small amplitude waves. However these edge filtering techniques are not easily multiplexable, which presents challenges when scaling to large structures. Therefore, there is a strong need for strategies to multiplex equipment for high frequency and low amplitude measurements. Multiplexing will not only allow for a reduction in the fibers deployed on the structure, but also reduce the instrumentation cost through the use of fewer laser sources.

Furthermore, OF-based sensors are mostly passive sensors, therefore they need additional actuation systems to generate GWs, except for acoustic emission applications. The need for actuators, for example piezo-electric devices, can undermine the advantages of the FO-based sensor systems. Some initial work has been conducted on the actuation of the GWs through optical fibers [170,171]. However, at the present moment, the actuation amplitudes are not as comparable as those achieved by piezo-based systems. Work in this direction would go a long way in replacing piezo-based systems in real applications.

In addition to offering the abilities of multiplexing and actuation, more developments at the sensor level and system level are necessary before the OF-based systems will be accepted in industrial applications. Novel sensor configurations continue to emerge frequently, but they remain at the lower end of the technology readiness level and their advantages or disadvantages for GW detection have not been explored. For example, tilted FBG sensors offer easy temperature compensation and may be interrogated similar to conventional FBG sensors [172]. Another promising area is the use of polarization maintaining (PM) FBG sensors. The slow and the fast axes of PM fibers have different sensitivities to GWs propagating in different directions [173] and may enable GW mode separation. In addition, the two axes may be used for temperature compensation. Similar arguments could be made for the use of photonic crystal fibers, as they can be fabricated to have significantly different wave speeds for the slow and fast axes. These fibers have been widely applied for strain and gas sensing. Wideband acoustic detection has been performed using photonic crystal slabs [174], so there is the potential to transition this technology to fiber sensors.

As discussed earlier, the coupling of GWs to optical fibers and their interactions is a complex phenomenon which depends on the materials at the interface as well as the bonding or contact conditions. In order to realistically understand this complex phenomenon, in depth numerical studies are necessary. Numerical modeling of these interactions remains computationally expensive due to the different time and space scales involved. Computationally efficient methods of simulation, such as the use of time and frequency domain spectral finite element methods with higher order shape functions, reducing the number of nodes and elements, need to be applied to these problems. These simulations could provide additional insights into the physics of the sensor response and methods to enhance their sensitivity to GWs.

Another area with potential to reduce the cost of the system is through the more efficient use of the resources. This can be achieved through the optimization of the sensor placement. Optimization methods have been developed for piezo-based systems [175], however, these methods cannot be applied directly to optical fiber sensors due to their passive nature and directional sensitivity. Soman et al. [113] developed an optimization system for FBG sensors based on genetic algorithm, which takes into consideration the directional sensitivity of the fibers. They later extended the work for the optimization of the actuators for a given location of the sensor [176,177]. However, in reality, the optimization of both the sensors and the actuators needs to be conducted jointly [178]. Different cost functions for the optimization, such as the probability of detection and coverage of the network, need to be implemented, keeping in mind the key goals of the SHM system that is to be designed.

## 6. Conclusions

The review paper gives an overview of the development in optical fiber based ultrasonic sensing systems. It introduces the operating principles of the different sensors used for the measurement of the ultrasonic waves. It then delves into the complex phenomena of the wave coupling from the structure to the fiber and then the propagation along the fiber. It discusses the different challenges the OF systems face for their use for SHM of structures. At the present time the use of FBG sensors for GW and AE based SHM is more dominant than all other sensor systems together. As a result a section was dedicated on the measurements using FBG sensors. The paper then highlights the several landmark papers where novel sensor techniques have been successfully used on structures. The paper builds on the previous review by Yu et al. and presents the new developments in SHM using FBG sensors which have occurred after the previous review paper. In the end, the paper points to the areas which need to be developed further for more widespread acceptance of optical fiber based ultrasonic sensing systems.

## Figures and Tables

**Table 1:** Summary of different sensor configurations, their applicability, and key indicators.

Sensor and Configuration	Imp	AE	AU	M	S	R	C
Polarimetric sensor	×	✓	✓	×	low	high	low
Microbend sensors	×	✓	×	×	low	high	low
Fused tapered coupler	×	✓	×	✓	high	low	low
FPI based sensors	✓	✓	✓	×	high	low	medium
MZI based sensors	✓	✓	✓	×	high	low	medium
FOD sensors	✓	✓	✓	×	medium	low	low
FBG in WDM configuration	✓	✓	×	✓	low	medium	medium
FBG in edge filtering configuration	✓	✓	✓	×	high	medium	high
MRR sensor	✓	✓	✓	×	high	low	high

Where, Imp—Impact, AE—acoustic emission, AU—acousto-ultrasonic, M—multiplexability, S—sensitivity, R—robustness, and C—cost.

## Data Availability

No new data were created or analyzed in this study. Data sharing is not applicable to this article.
